# Hedgehog Acyltransferase as a target in estrogen receptor positive, HER2 amplified, and tamoxifen resistant breast cancer cells

**DOI:** 10.1186/s12943-015-0345-x

**Published:** 2015-04-01

**Authors:** Armine Matevossian, Marilyn D Resh

**Affiliations:** Cell Biology Program, Memorial Sloan Kettering Cancer Center, 1275 York Avenue, Box 143, New York, NY 10065 USA; Gerstner Sloan Kettering Graduate School of Biomedical Sciences, Memorial Sloan Kettering Cancer Center, New York, NY USA

**Keywords:** Breast cancer, Hedgehog, Hedgehog acyltransferase, Tamoxifen resistant, PI3K/mTOR

## Abstract

**Background:**

Hedgehog acyltransferase (Hhat) catalyzes the transfer of the fatty acid palmitate onto Sonic Hedgehog (Shh), a modification that is essential for Shh signaling activity. The Shh signaling pathway has been implicated in the progression of breast cancer.

**Methods:**

To determine the functional significance of Hhat expression in breast cancer, we used a panel of breast cancer cell lines that included estrogen receptor (ER) positive, HER2 amplified, triple negative, and tamoxifen resistant cells. We monitored both anchorage dependent and independent proliferation of these cells following depletion of Hhat with lentiviral shRNA and inhibition of Hhat activity with RU-SKI 43, a small molecule inhibitor of Hhat.

**Results:**

Depletion of Hhat decreased anchorage-dependent and anchorage-independent proliferation of ER positive, but not triple negative, breast cancer cells. Treatment with RU-SKI 43 also reduced ER positive cell proliferation, whereas a structurally related, inactive compound had no effect. Overexpression of Hhat in ER positive cells not only rescued the growth defect in the presence of RU-SKI 43 but also resulted in increased cell proliferation in the absence of drug. Furthermore, depletion or inhibition of Hhat reduced proliferation of HER2 amplified as well as tamoxifen resistant cells. Inhibition of Smoothened had no effect on proliferation, indicating that canonical Shh signaling was not operative. Moreover, Hhat regulated the proliferation of both Shh responsive and non-responsive ER positive cells, suggesting a Shh independent function for Hhat.

**Conclusions:**

These data suggest that Hhat plays a critical role in ER positive, HER2 amplified, and hormone resistant breast cancer proliferation and highlights the potential promise of Hhat inhibitors for therapeutic benefit in breast cancer.

**Electronic supplementary material:**

The online version of this article (doi:10.1186/s12943-015-0345-x) contains supplementary material, which is available to authorized users.

## Background

Breast cancer is the most common cancer affecting women [1]. Gene expression profiling has identified distinct biological subtypes of breast cancer: luminal A or B, human epidermal growth factor receptor 2 (HER2) amplified, basal like, and claudin low [2]. The luminal A and B subtypes are both estrogen receptor (ER) positive and comprise up to 70% of all breast cancers. Luminal B tumors are also HER2 positive and have a poorer prognosis [2-4]. The basal like and claudin low subtypes are both triple negative, lacking expression of ER, HER2 and the progesterone receptor. Treatment of luminal A tumors with tamoxifen, a selective ER modulator, has significantly reduced the mortality rate. However, not all patients respond to tamoxifen and one third of initial responders have recurrent disease within 15 years [5]. Hormone resistance can occur through ER-dependent as well as ER-independent mechanisms, including activation of pro-proliferative signaling pathways such as HER2 and EGFR [6], PI3K/Akt, and MAPK [7]. Use of trastuzumab, an antibody targeting HER2, has extended the overall survival of patients with HER2 amplified tumors [8]. However, about 40-60% of these tumors show *de novo* resistance even when treatment is combined with systematic chemotherapy [9]. Furthermore, about 70% of initial responders show progressive disease within a year. Acquired resistance can occur through overexpression of EGFR family receptors [10] or IGF-R1 [11], PTEN loss, or activation of PI3KCA [12,13]. Therefore, there is a need to identify new therapeutic targets.

Recently, aberrant activation of the Sonic Hedgehog (Shh) pathway has been implicated in breast cancer progression [14-26]. The hedgehog family of secreted signaling molecules includes Shh, Indian and Desert Hedgehog. Interaction of Shh with the transmembrane receptor Patched-1 (Ptch-1) relieves inhibition of the transducer Smoothened (Smo). This leads to the stabilization and nuclear translocation of the Gli family of transcription factors [27]. The resulting activation of target gene transcription regulates various cellular processes such as cell fate determination, proliferation, and survival [27]. A role for abnormal Shh signaling activity in breast cancer development was first reported using transgenic mouse models, where Ptch-1 haploinsufficiency or ectopic expression of Smo lead to distinct forms of mammary ductal dysplasia [28,29]. Furthermore, expression of Gli-1 under the mouse mammary tumor virus promoter leads to the development of hyperplastic lesions and tumors [22]. Mutations in Shh, Ptch, and Smo are rarely identified in human breast cancer [23]. Ptch expression is reduced in ductal carcinoma *in situ* (DCIS) [29,30], possibly due to increased promoter methylation [30]. In addition, ectopic expression of Smo has been identified in both DCIS and invasive breast cancer [29]. Breast tumor growth and metastasis in mice is stimulated by Shh overexpression and is decreased by inhibiting Shh signaling [14]. In humans, Shh overexpression occurs in breast tumor initiating cells and in invasive ductal carcinoma (IDC), where it is associated with increased metastasis and death [14]. A progressive increase in Shh expression correlates with disease progression from low grade DCIS to IDC [14,15]. In addition, three studies have noted strong Gli-1 expression in stromal cells [14,18,19]. Shh and Ihh secreted by breast cancer cells can signal in a paracrine manner to induce osteoclast differentiation and increase bone resorption [24]. Furthermore, other pathways, including osteopontin and TGFβ, can also activate Gli-mediated transcription in breast cancer cells [25,26].

To date, analyses of the hedgehog pathway in breast cancer have focused mainly on downstream signaling events. Little is known about components of the pathway upstream of ligand production. Shh is synthesized as a precursor protein that undergoes autoprocessing to produce a ~25 kDa C-terminal fragment and a ~19 kDa N-terminal fragment (ShhN) that retains all signaling activity [31,32]. ShhN is modified with two lipids. Cholesterol is covalently attached to the C-terminus during the autoprocessing reaction [33]. Cholesterol attachment contributes to long-range signaling activity, but is not essential for signaling [34]. The N-terminus of ShhN is modified by covalent attachment of the 16-carbon fatty acid palmitate to the N-terminal cysteine [35,36]. Shh palmitoylation is catalyzed by Hedgehog acyltransferase (Hhat), a multipass transmembrane enzyme that belongs to the membrane bound O-acyltransferase (MBOAT) family [36]. Multiple studies have established that palmitoylation of Shh by Hhat is critical for Shh signaling activity [34,37-40]. Furthermore, Hhat activity is required for the proliferation of pancreatic cancer cells *in vivo* and for the maintenance of a stem-like phenotype in lung squamous cell carcinoma [41-44].

The role of Hhat in breast cancer has not yet been examined. In this study, we demonstrate that Hhat is required for the proliferation of ER positive, HER2 positive, and tamoxifen resistant breast cancer cells. Increased Hhat expression resulted in increased cell proliferation, while Hhat depletion reduced proliferation of ER positive cells. Hhat inhibition with RU-SKI 43, a selective small molecule inhibitor of Hhat recently identified by our group [45], also reduced the growth of ER positive cells. Furthermore, Hhat depletion or inhibition led to a significant decrease in HER2 positive and tamoxifen resistant cell proliferation. None of the cell lines we tested responded to inhibition of Smo, and only a subset responded to Shh depletion, indicating that non-canonical Shh signaling pathways were operative. Taken together, these data suggest that Hhat may serve as an important therapeutic target in ER positive, HER2 amplified, and hormone resistant breast cancers.

## Results

### Hhat depletion results in reduced ER positive breast cancer cell proliferation

**Figure 1:**
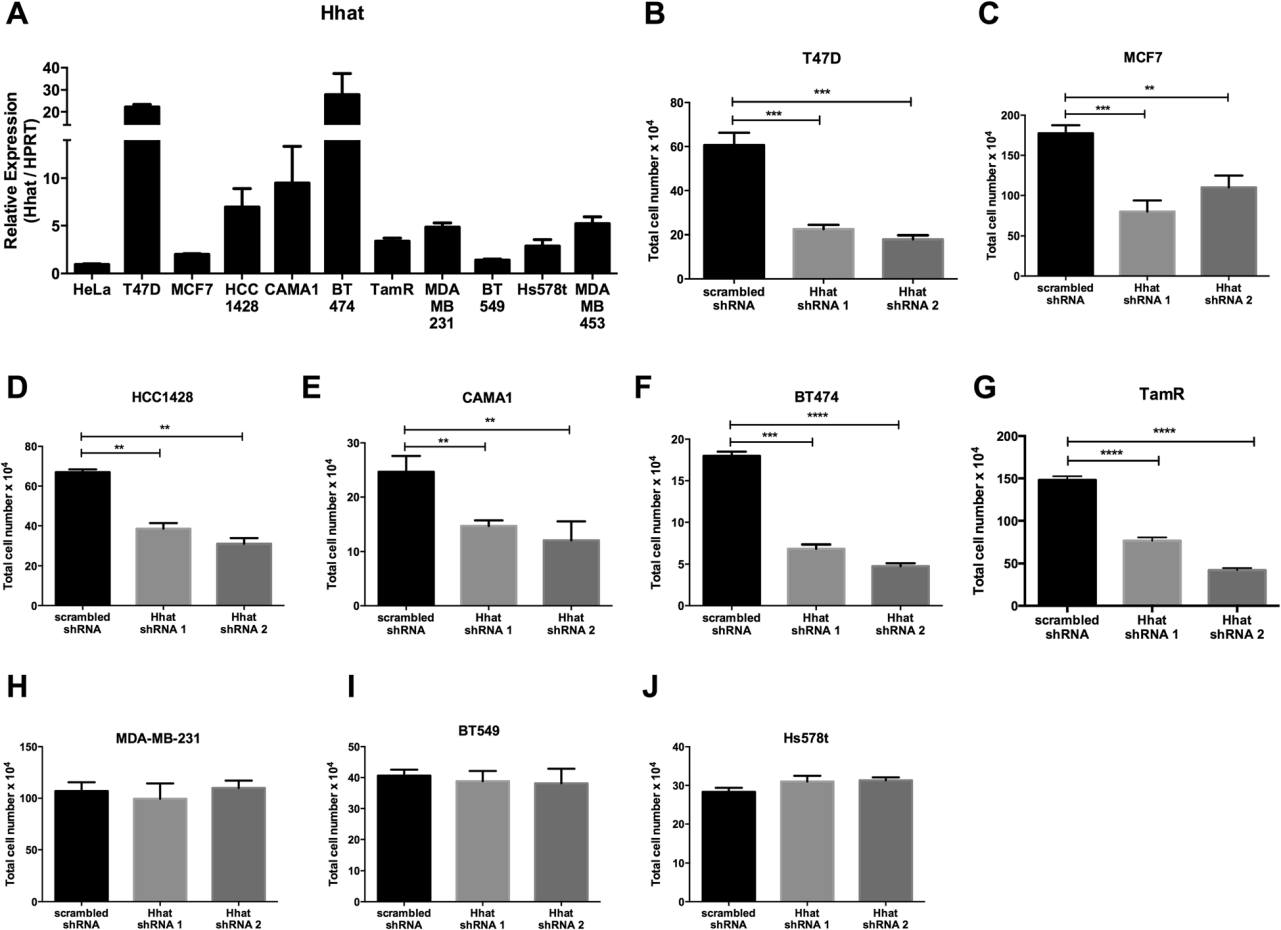
**Hhat depletion reduces proliferation of ER positive breast cancer cells. A**, Hhat mRNA expression in indicated breast cancer cell lines and a control cervical cancer (HeLa) cell line, was measured by qRT-PCR. Hhat expression is shown relative to the expression in HeLa cells, which is set to 1. Bars represent mean ± SD (n = 3). Experiments were performed twice in triplicate. **B-J**, Total cell number at day 6 for **(B)** T47D, **(C)** MCF7, **(D)** HCC1428, **(E)** CAMA-1, **(F)** BT474, **(G)** TamR, **(H)** MDA-MB-231, **(I)** BT549, and **(J)** Hs578t. Cells stably expressing scrambled or Hhat shRNAs were seeded at 5-7 × 10^4^ cells/well, depending on cell type, in 6-well plates and cell numbers were quantified on day 6. For panels **B-J**, Bars represent mean ± SD (n = 3). Three independent experiments were performed in duplicate using cells at three different passages. *P ≤ 0.05; **P ≤ 0.01; ***P ≤ 0.001; ****P ≤ 0.0001; Student’s *t* test.

**Figure 2:**
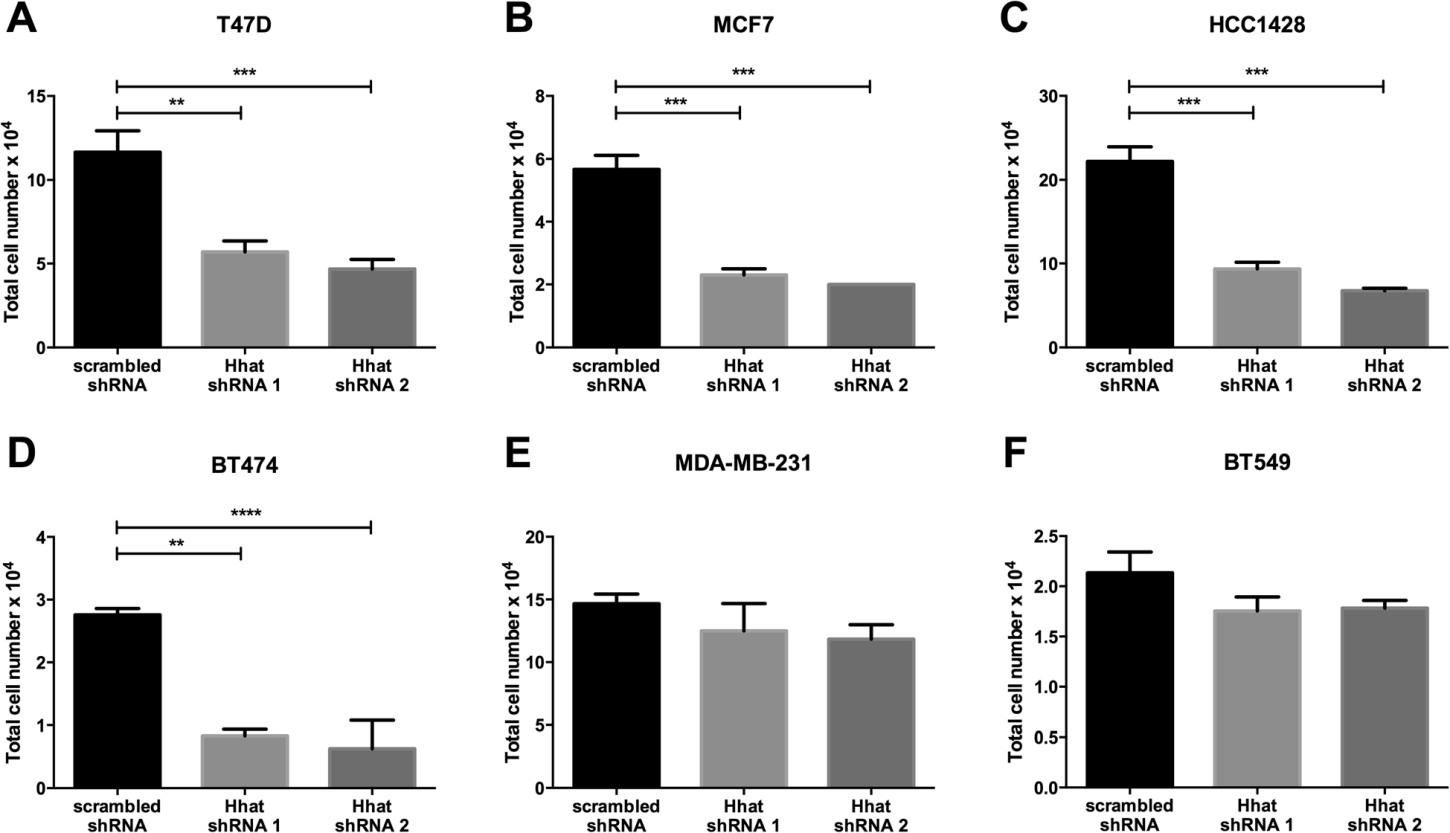
**Hhat depletion reduces anchorage independent proliferation of ER positive cells. A**-**F**, indicated breast cancer cell lines stably expressing scrambled or Hhat shRNAs were seeded at 1-2 × 10^4^ cells/well in 24-well ultra-low adherence plates, and cell numbers were quantified 14 days later. Bars represent mean ± SD (n = 3) for all panels. Three independent experiments were performed in duplicate using cells at three different passages. *P ≤ 0.05; **P ≤ 0.01; ***P ≤ 0.001; ****P ≤ 0.0001; Student’s *t* test.

To investigate the role of Hhat in breast cancer, we used a panel of ER positive (T47D, MCF7, HCC1428, CAMA-1, and BT474) and ER negative (MDA-MB-231, BT549, Hs578t, and MDA-MB-453) cell lines. ER and HER2 expression status was verified in the above cell lines (Additional file 1: Figure S1). Hhat mRNA was detected in all cell lines to varying degrees, with mostly higher expression in the ER positive cells (Figure 1A). To assess the functional significance of Hhat expression in breast cancer cells, two different lentiviral based short hairpin RNAs were used to stably deplete Hhat mRNA. Hhat depletion (Additional file 2: Figure S2A) led to a 66% reduction in proliferation of ER positive T47D cells, compared to the scrambled shRNA control (Figure 1B). Similar results were observed in all ER positive cell lines (Figure 1C-F, Additional file 2: Figure S2B-E). By contrast, depletion of Hhat in triple negative cells (Additional file 2: Figure S2G-I) did not alter cell proliferation (Figure 1H-J). We then monitored anchorage independent growth, a hallmark of neoplastic cells. Hhat depletion in ER positive, but not in triple negative, cells resulted in markedly reduced anchorage independent proliferation (Figure 2A-F). These data indicate that Hhat regulates anchorage dependent and independent proliferation of ER positive cells.

### Hhat inhibition leads to decreased ER positive breast cancer cell proliferation

**Figure 3:**
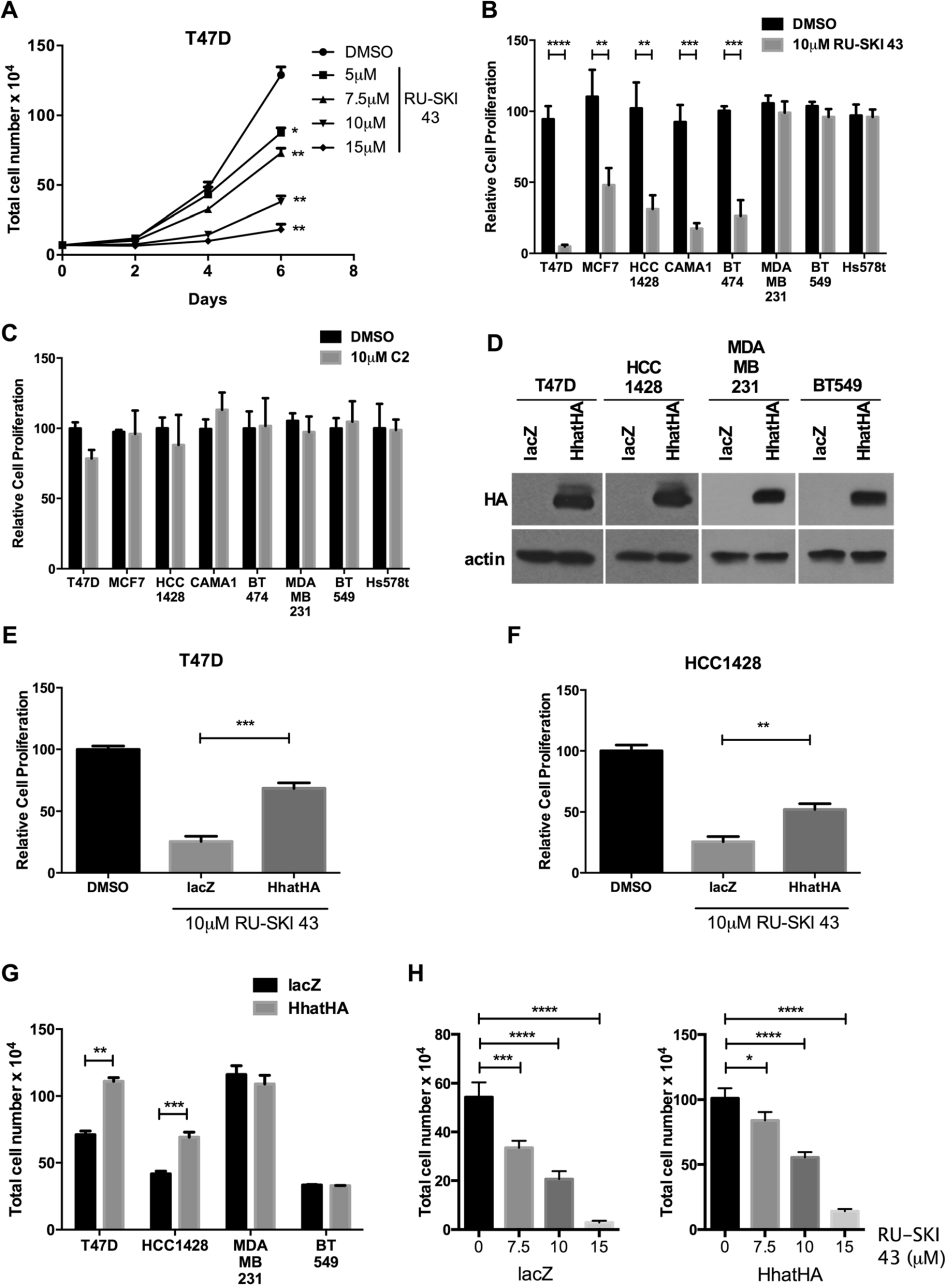
**Hhat inhibition with RU-SKI 43 results in decreased proliferation of ER positive cells. A**, T47D cells were seeded at 7 × 10^4^ cells/well in 6-well plates. 24 hrs post seeding, cells were treated with either DMSO or the indicated concentrations of RU-SKI 43. Media was changed every 48 hrs and cell numbers were quantified 2, 4, and 6 days post treatment. **B**, indicated cell lines were seeded at 5-7 × 10^4^ cells/well in 6-well plates. 24 hrs post seeding, cells were treated with either DMSO or 10 μM RU-SKI 43. Cell numbers were quantified 6 days post treatment and expressed relative to growth in DMSO (100 x (RU-SKI 43/DMSO)). **C**, indicated cell lines were seeded at 5-7 × 10^4^ cells/well in 6-well plates. 24 hrs post seeding, cells were treated with either DMSO or 10 μM C2. Cell numbers were quantified 6 days post treatment and expressed relative to growth in DMSO. **D**, cell lysates from T47D, HCC1428, MDA-MB-231, and BT549 cells stably expressing LacZ or HhatHA were analyzed directly by Western blotting. **E**-**F**, **(E)** T47D and (F) HCC1428 cells stably expressing LacZ or HhatHA were seeded at 7 × 10^4^ cells/well in 6-well plates and grown in media containing DMSO or 10 μM RU-SKI 43. Cell numbers were quantified on day 6 and expressed relative to DMSO treated cells. The increase in proliferation between Hhat and LacZ overexpressing cells in the presence of RU-SKI 43 is 176% and 106%, for T47D and HCC1428 respectively. **G**, growth curves for T47D, HCC1428, MDA-MB-231, and BT549 cells stably expressing LacZ or HhatHA. Cells were seeded at 5-7 × 10^4^ cells/well and cell numbers were quantified on day 6. The increase in proliferation in response to overexpressing Hhat in untreated cells is 56% and 61%, for T47D and HCC1428 respectively. **H**, T47D cells overexpressing lacZ or HhatHA were cultured in the presence of DMSO or the indicated concentrations of RU-SKI 43. Cells numbers were quantified on day 6. Bars represent mean ± SD (n = 3) for all panels. Three independent experiments were performed in duplicate using cells at three different passages. *P ≤ 0.05; **P ≤ 0.01; ***P ≤ 0.001; ****P ≤ 0.0001; Student’s *t* test.

To validate that Hhat activity is required for ER positive breast cancer cell growth, we used RU-SKI 43, a selective small molecule inhibitor of Hhat previously identified by our laboratory [45]. Treatment of T47D cells, which express relatively high levels of Hhat, with increasing concentrations of RU-SKI 43 resulted in a dose dependent decrease in cell proliferation (Figure 3A). Moreover, Hhat inhibition also significantly reduced proliferation of all ER positive cells tested (56-95% depending on cell type) but had no effect on triple negative cells (Figure 3B). Importantly, C2, a compound that is structurally related to RU-SKI 43 but does not inhibit Hhat activity [45], did not affect breast cancer cell proliferation (Figure 3C). The growth defect induced by RU-SKI 43 was rescued, in part, by Hhat overexpression (Figure 3D-F). These data indicate that Hhat inhibition by RU-SKI 43 reduces ER positive cell proliferation.

### Hhat overexpression results in increased proliferation of ER positive cells

We next performed a gain of function experiment by testing the effect of Hhat overexpression. Stable lines of ER positive (T47D, HCC1428) and ER negative (MDA-MB-231, BT549) cells expressing either control LacZ or Hhat (Figure 3D) were generated. T47D and HCC1428 cells expressing Hhat exhibited 56% and 61% increases, respectively, in cell proliferation compared to control cells expressing LacZ, while overexpression of Hhat in ER negative cells had no effect on cell proliferation (Figure 3G). These findings indicate that increased Hhat activity can enhance ER positive cell proliferation. We then compared the response of cells stably expressing LacZ or Hhat to increasing concentrations of RU-SKI 43. Hhat overexpression blunted the inhibitory effect of RU-SKI 43 on cell proliferation (Figure 3H), supporting the hypothesis that the effect of RU-SKI 43 is mainly due to inhibition of Hhat.

### RU-SKI 43 does not alter ERα localization or activation

**Figure 4:**
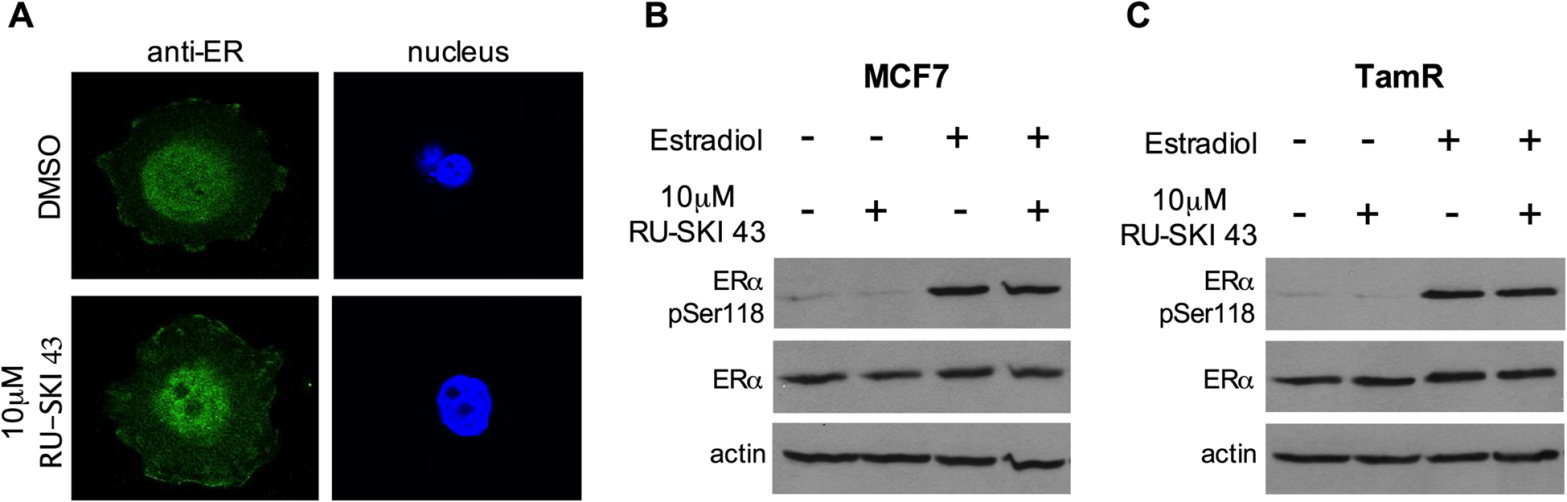
**RU-SKI 43 does not alter localization or activation of ERα. A**, MCF7 cells were cultured in the presence of DSMO or 10 μM RU-SKI 43 for 4 h. Cells were fixed and stained with anti-ERα. Three independent experiments were performed using cells at three different passages. **B**-**C**, MCF7 **(B)** or TamR **(C)** cells were treated with DSMO or 10 μM RU-SKI 43 for 4 h. Cells were treated with ethanol or 17β-estradiol for 30 minutes prior to lysis. Cell lysates were analyzed directly by Western blotting with indicated antibodies. Three independent experiments were performed in duplicate using cells at three different passages.

To examine whether Hhat functions through an ERα-dependent mechanism, we examined the effects of Hhat inhibition on ERα palmitoylation, localization, and activation. ERα has been reported to be palmitoylated, and palmitoylation has been proposed to mediate localization of a subpopulation of ERα to the plasma membrane [46,47]. We used ^125^I-iodopalmitate, a radioiodinated palmitate analog that allows for sensitive and robust detection of palmitoylated proteins in cells [45]. However, we were unable to detect incorporation of ^125^I-iodopalmitate into either endogenous or overexpressed ERα in MCF7 cells. To determine whether RU-SKI 43 affects ERα localization to the plasma membrane, the subcellular localization of endogenous ERα was compared in MCF7 cells treated with either DMSO or RU-SKI 43. ERα localized to the nucleus, cytoplasm, and plasma membrane, consistent with previous reports [46,47], and treatment with RU-SKI 43 did not alter the ERα localization pattern (Figure 4A). Finally, the ability of estradiol to induce phosphorylation of ERα at Ser118, a marker of receptor activation [48,49], was not altered by treatment with RU-SKI 43 (Figure 4B). These data indicate that the effect of the Hhat inhibitor RU-SKI 43 on ER positive cell proliferation is not due to a direct modulation of ERα localization or activation.

### Non-canonical Shh signaling regulates proliferation of a subset of breast cancer cells

**Figure 5:**
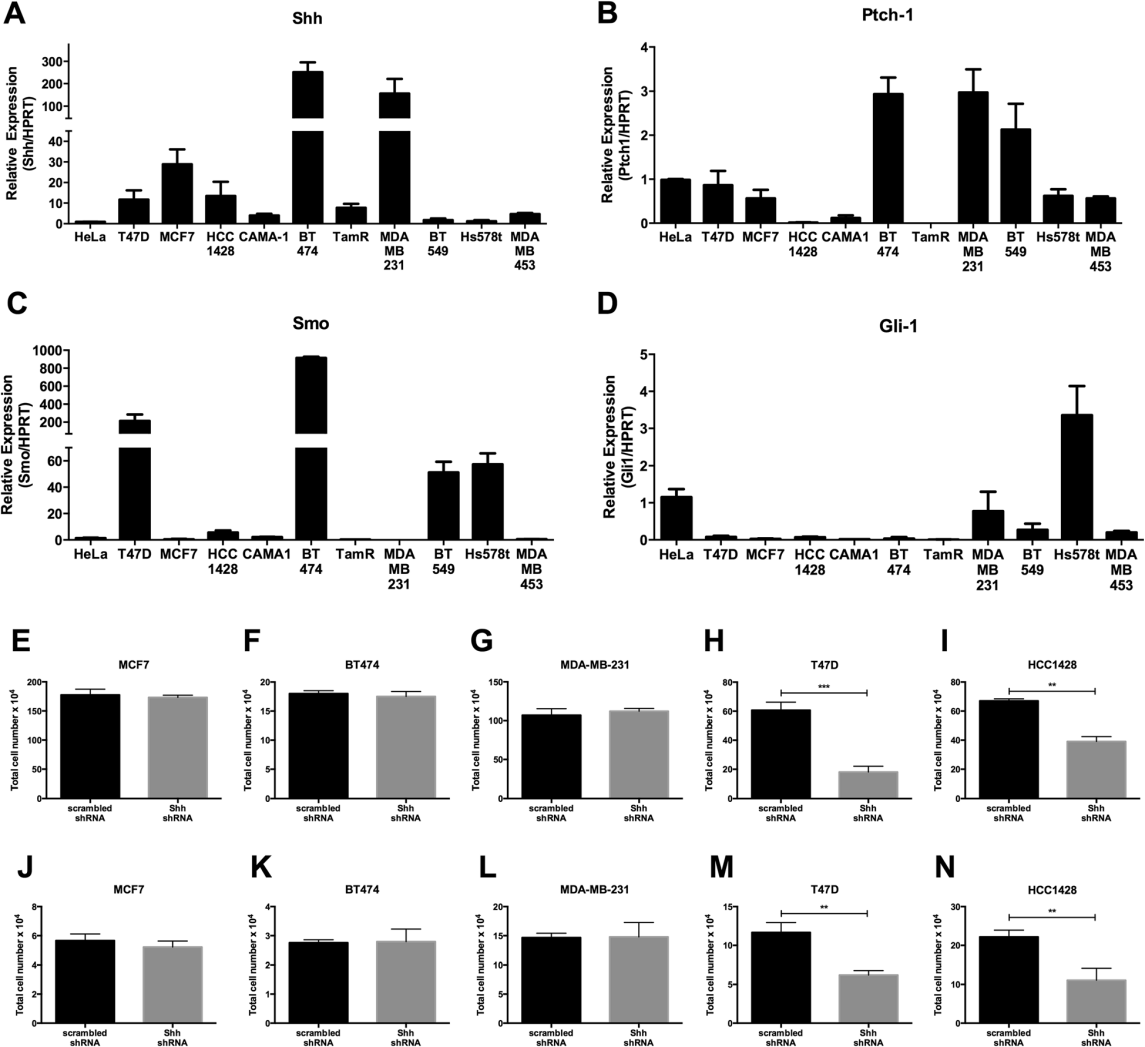
**Analysis of Shh signaling pathway components in breast cancer cells. A**-**D**, expression of **(A)** Shh, **(B)** Ptch-1, **(C)** Smo, and **(D)** Gli-1 mRNAs in indicated breast cancer cell lines and a control cervical cancer (HeLa) cell line, was measured by qRT-PCR. Expression of individual genes is shown relative to the expression in HeLa cells, which is set to 1. Bars represent mean ± SD (n = 3). Experiments were performed twice in triplicate. **E**-**I**, indicated breast cancer cells stably expressing scrambled or Shh shRNAs were seeded at 5-7 × 10^4^ cells/well, depending on cell type, in 6-well plates and cell numbers were quantified on day 6. **J**-**N**, indicated breast cancer cell lines stably expressing scrambled or Shh shRNAs were seeded at 1-2 × 10^4^ cells/well in 24-well ultra-low adherence plates, and cell numbers were quantified 14 days later. For E-N, bars represent mean ± SD (n = 3). Three independent experiments were performed in duplicate using cells at three different passages. *P ≤ 0.05; **P ≤ 0.01; ***P ≤ 0.001; ****P ≤ 0.0001; Student’s *t* test.

Hhat is the palmitoyl acyltransferase for the hedgehog family of proteins and is required for efficient Shh signaling [36,40]. Therefore, we examined whether the effect of Hhat on proliferation is mediated through hedgehog signaling. First, we quantified the expression of hedgehog pathway components in breast cancer cells. Shh mRNA was expressed in T47D, MCF7, HCC1428, BT474, and MDA-MB-231 cells (Figure 5A). Ihh was detected in MDA-MB-231 and BT549 cells, and Dhh was detected in T47D and MDA-MB-231 cells (Additional file 3: Figure S3A, B). Ptch-1 and Ptch-2 expression was detectable in nearly all cells (Figure 5B, Additional file 3: Figure S3C). Although Smo was expressed in T47D, BT474, and BT549 cells (Figure 5C), little to no Gli-1 or Gli-2 was expressed in these cells (Figure 5D, Additional file 3: Figure S3D), suggesting either a cell non-autonomous or non-canonical role for Shh. Hs578t, which does not respond to Hhat depletion or inhibition (Figures 1J and 3B), was the only cell line that expressed both Smo and Gli-1 (Figure 5C,D). Repressors of the Shh pathway were only detected in a few cell lines (hHIP) or at very low levels (Gli-3) (Additional file 3: Figure S3E, F).

Several breast cancer cell lines (T47D, MCF7, HCC1428, BT474, and MDA-MB-231) express high levels of Shh (Figure 5A). To test whether the growth of these cell lines was dependent on Shh, stable expression of Shh targeting shRNAs was used to reduce Shh levels (Additional file 4: Figure S4A-E). No effect of Shh depletion was observed on either anchorage dependent or independent growth in MCF7, BT474, and MDA-MB-231 cells (Figure 5E-G, J-L). To investigate whether the lack of response to Shh knockdown in these three cell lines was due to upregulation of other hedgehog ligands, levels of Ihh and Dhh were quantified in Shh depleted cells. Neither Ihh nor Dhh were detected in MCF7 and BT474 (Ct values above 35) in either scrambled control or Shh knockdown cells. In MDA-MB-231 cells, Ihh and Dhh expression was detected but did not increase after Shh knockdown (Additional file 4: Figure S4F, G). These data indicate that certain ER positive cells require Hhat but not Shh for proliferation, suggesting a Shh independent role for Hhat.

**Figure 6:**
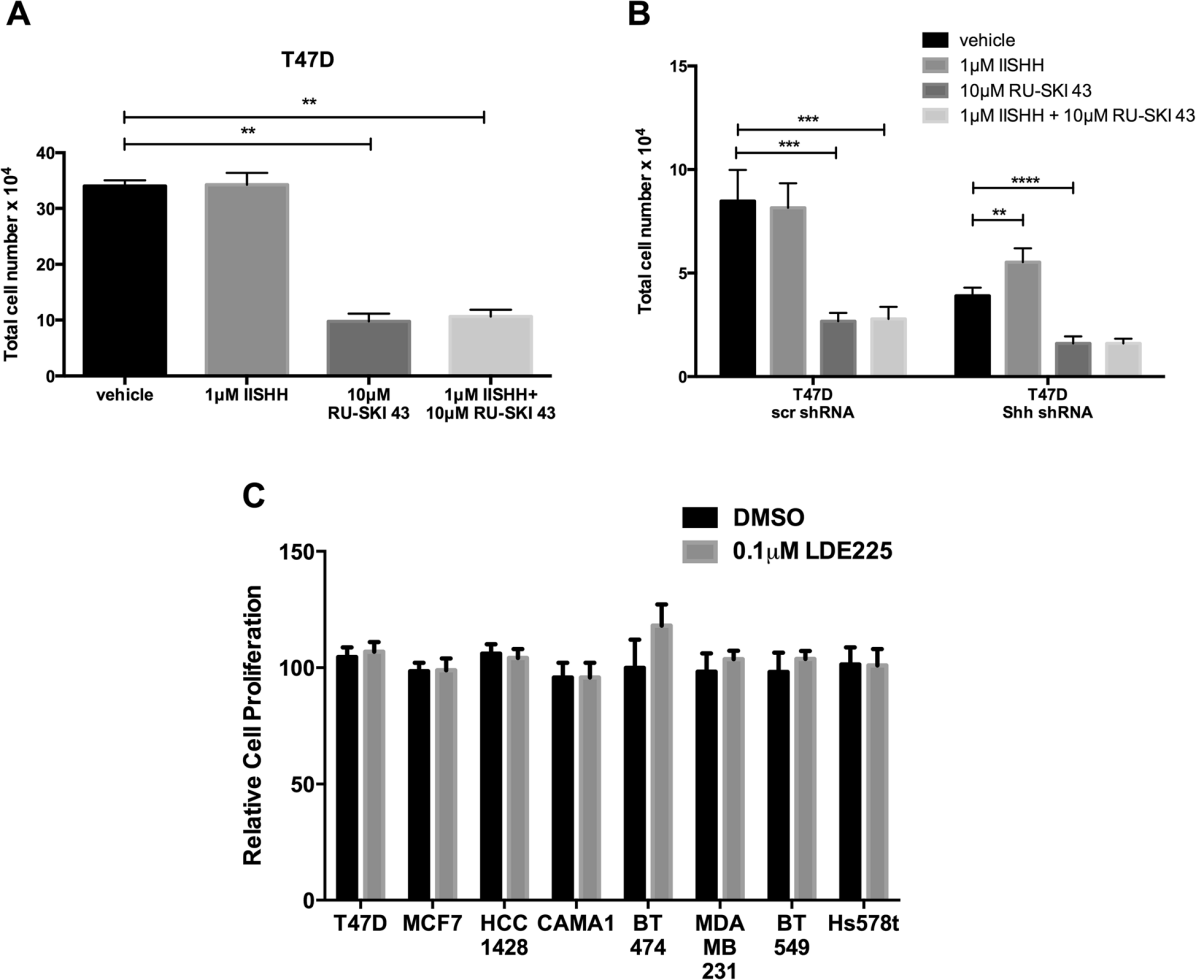
**Evidence for non-canonical Shh signaling in breast cancer cells. A**, T47D cells were cultured in the presence of 1 μM Shh(CII24), 10 μM RU-SKI 43, or both for 6 days. Cell numbers were quantified and normalized to vehicle treated cells (100 x (drug/vehicle)). Bars represent mean ± SD (n = 3). Experiments were performed twice in triplicate. *P ≤ 0.05; **P ≤ 0.01; ***P ≤ 0.001; ****P ≤ 0.0001; Student’s *t* test. **B**, T47D cells were transduced with either a control scrambled or Shh shRNA expressing lentivirus and selected in puromycin. Cells were then cultured in the presence of 1 μM Shh(CII24), 10 μM RU-SKI 43, or both for 6 days. Cell numbers were quantified and normalized to vehicle treated cells (100 x (drug/vehicle)). Bars represent mean ± SD (n = 3). Experiments were performed twice in triplicate. **P ≤ 0.01; ***P ≤ 0.001; ****P ≤ 0.0001; Student’s *t* test. **C**, indicated cell lines were seeded at 5-7 × 10^4^ cells/well in 6-well plates. 24 hrs post seeding, cells were treated with either DMSO or 0.1 μM LDE225. Cell numbers were quantified 6 days post treatment and expressed relative to growth in DMSO. Bars represent mean ± SD (n = 3). Three independent experiments were performed in triplicate using cells at three different passages.

We identified two cell lines, T47D and HCC1428, in which Shh depletion reduced both anchorage-dependent (Figure 5H,I) and anchorage-independent proliferation (Figure 5M,N). We next asked whether decreased Shh signaling was responsible for the reduction in cell proliferation observed upon Hhat inhibition. If RU-SKI 43 reduces cell proliferation through Shh, then addition of exogenous, recombinant Shh(C24II) should rescue the growth of these cells in the presence of RU-SKI 43. When Shh(C24II) was added to T47D cells, no effect on cell proliferation was observed (Figure 6A). However, we and others have previously shown that in Shh producing cells, the hedgehog signaling machinery is saturated and a response to exogenous Shh is only revealed after endogenous Shh depletion [41,50,51]. Addition of Shh(C24II) rescued, in part, the growth defect of Shh-depleted T47D cells, but had no effect on T47D cells expressing the scrambled control shRNA (Figure 6B). However, treatment of Shh-depleted cells with RU-SKI 43 further decreased their growth, suggesting a role for Hhat in addition to Shh signaling (Figure 6B).

To examine whether canonical Smo-mediated Shh signaling is required for the proliferation of these cells, the effect of LDE-225, a Smo inhibitor, was analyzed. Nanomolar concentrations of LDE-225 inhibit canonical Shh signaling [52] and decrease the growth of LDE-225 sensitive tumor cells [53]. We used LDE-225 at 0.1 μM, a concentration 100x higher than IC_50_ for binding of LDE-225 to Smo [54], and found it had no effect on the proliferation of any of the breast cancer cell lines (Figure 6C), suggesting that Smo-mediated signaling is absent in these cells. This is consistent with our finding that T47D and HCC1428 cells have little to no Gli-1 expression (Figure 5D).

### Hhat depletion or inhibition reduces proliferation of HER2 amplified cells

**Figure 7:**
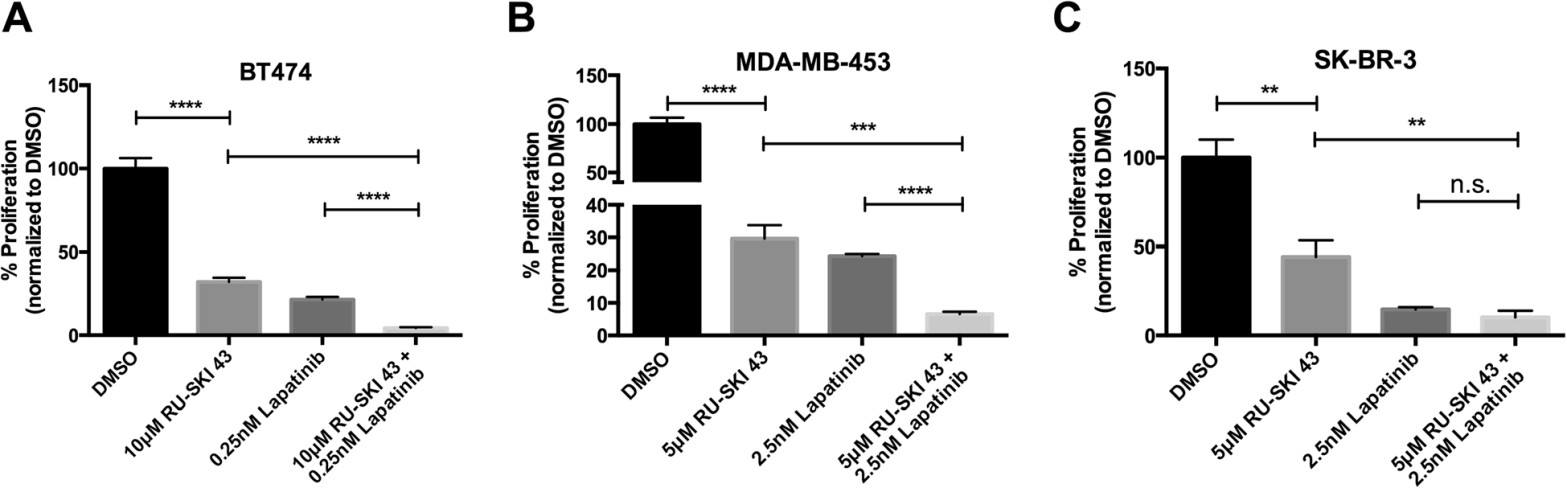
**Hhat inhibition reduces proliferation of HER2 amplified cells. A**-**C**, BT474 **(A)**, MDA-MB-453 **(B)**, and SK-BR3 **(C)** cells were cultured for 6 days in the presence of DMSO, RU-SKI 43 alone or in combination with indicated concentrations of lapatinib. Cell numbers were quantified and normalized to vehicle treated cells (100 x (drug/vehicle). Bars represent mean ± SD (n = 3) for all panels. Each experiment was performed using three separate passages of cells in triplicate. *P ≤ 0.05; **P ≤ 0.01; ***P ≤ 0.001; ****P ≤ 0.0001; Student’s *t* test.

ER positive/HER2 positive BT474 cells are sensitive to Hhat depletion or inhibition (Figures 1, 2, 3 and 7A). We therefore tested whether Hhat activity is also required for the growth of HER2 positive cells that are ER negative. Treatment of MDA-MB-453 and SK-BR-3 cells with RU-SKI 43 reduced proliferation, while C2 had no effect (Figure 7B,C, Additional file 5: Figure S5A). Depletion of Hhat in MDA-MB-453 cells (Additional file 5: Figure S5B) also led to a significant reduction in proliferation (Additional file 5: Figure S5C). Thus, Hhat activity is required for the proliferation of HER2 amplified cells independently of ER status. Furthermore, inhibition of both Hhat and HER2 by combined treatment with RU-SKI 43 and lapatinib resulted in significantly reduced proliferation of BT474 and MDA-MB-453 cells when compared to treatment with either agent alone (Figure 7A,B). Taken together, these data suggest that Hhat inhibition may be combined with current HER2 targeted therapies to achieve a more potent inhibition of breast cancer cell proliferation.

### Combined inhibition of Hhat and PI3K/mTOR effectively reduces breast cancer cell proliferation

**Figure 8:**
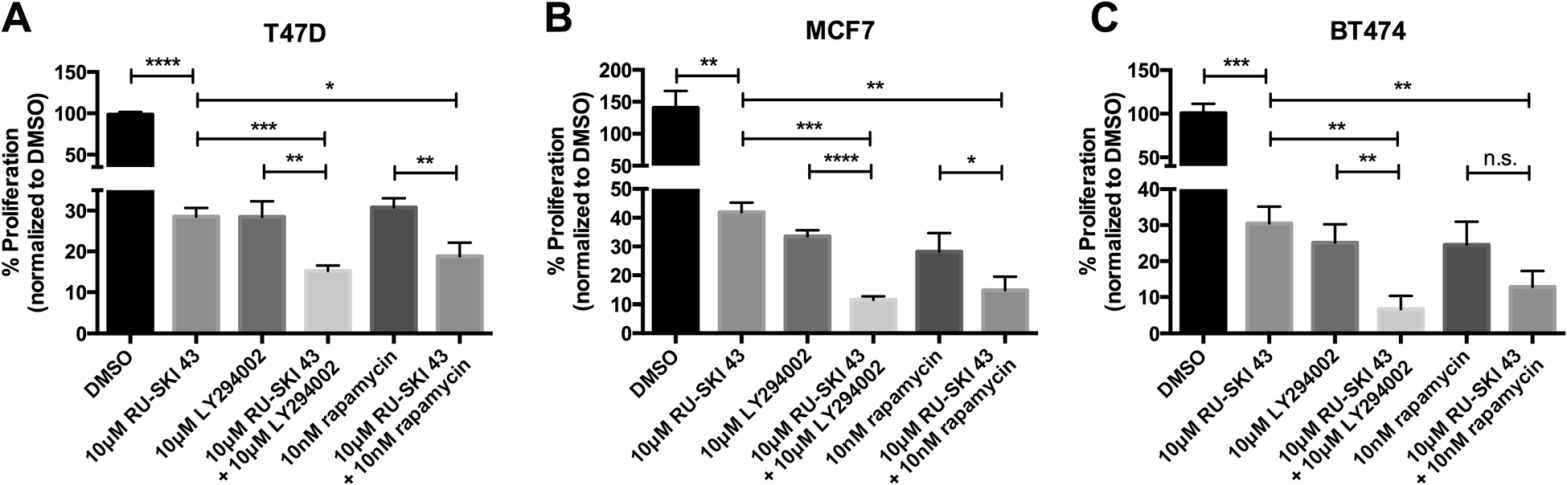
**Combined inhibition of Hhat and PI3K/mTOR effectively reduces breast cancer cell proliferation. A**-**C**, T47D **(A)**, MCF7 **(B)**, and BT474 **(C)** cells were cultured for 6 days in the presence of 10 μM RU-SKI 43 alone or in combination with 10 μM LY294002 or 10nM rapamycin. Cell numbers were quantified and normalized to DMSO treated cells (100 x (drug/DMSO)). Bars represent mean ± SD (n = 3) for all panels. Three independent experiments were performed in duplicate using cells at three different passages. *P ≤ 0.05; **P ≤ 0.01; ***P ≤ 0.001; ****P ≤ 0.0001; Student’s *t* test.

Activation of PI3K/mTOR signaling occurs in up to a quarter of both ER positive and HER2 positive breast cancers [13] and several inhibitors are currently in clinical trials [55]. Furthermore, increased signaling through this pathway is also associated with resistance to available therapies [13]. Therefore, we next examined whether RU-SKI 43 could be effectively combined with PI3K or mTOR inhibitors to reduce cell proliferation. Combined treatment of ER positive breast cancer cells with RU-SKI 43 and either the PI3K inhibitor LY294002 or the mTOR inhibitor rapamycin resulted in a further decrease in cell proliferation compared to either drug alone (Figure 8A-C). Thus, simultaneous inhibition of Hhat and PI3K/mTOR signaling effectively reduces breast cancer cell proliferation.

### Hhat depletion or inhibition reduces proliferation of tamoxifen resistant cells

**Figure 9:**
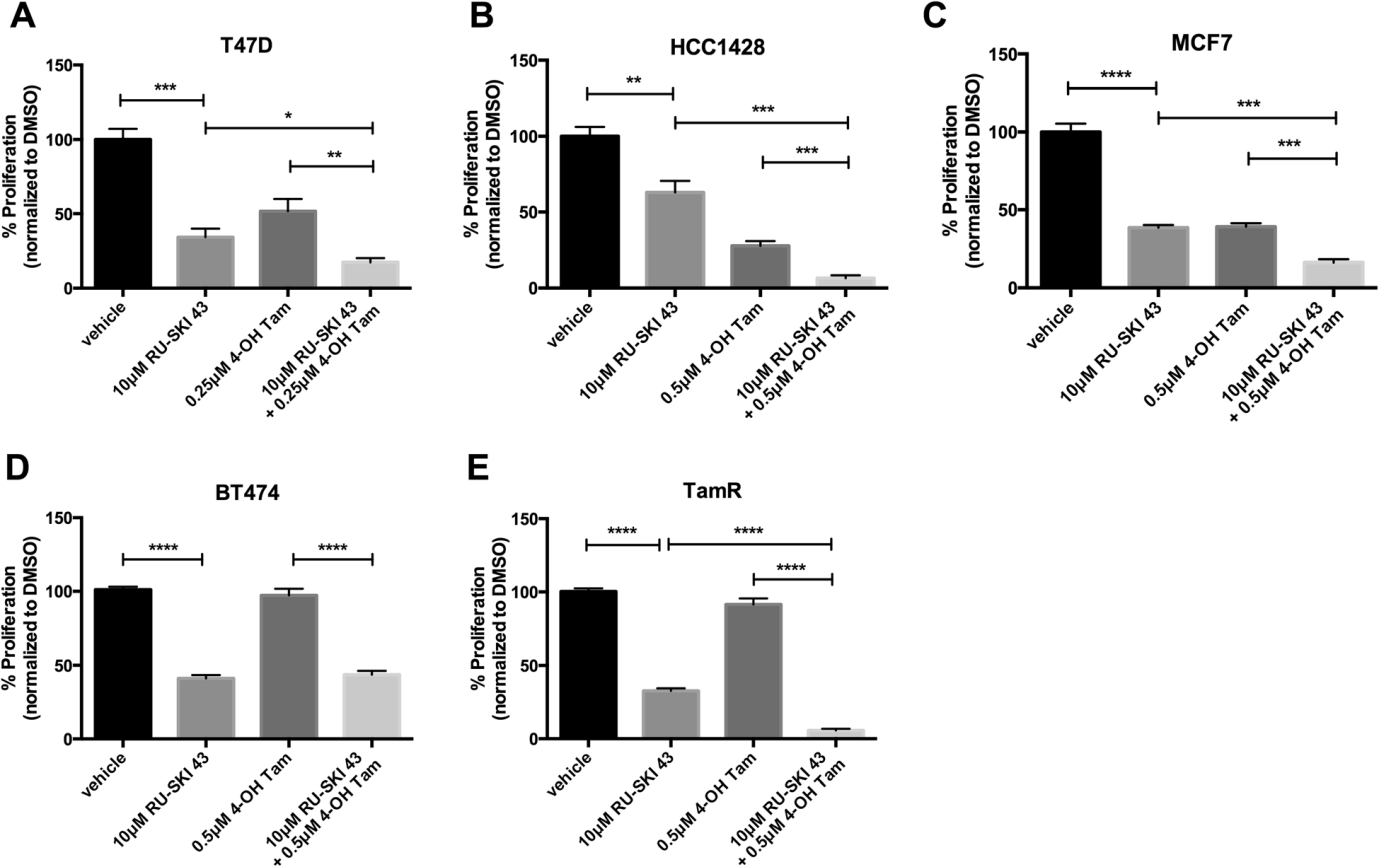
**Tamoxifen resistant cells are sensitive to Hhat inhibition. A**-**D**, T47D **(A)**, HCC1428 **(B)**, MCF7 **(C)**, BT474 **(D)**, and TamR **(E)** cells were cultured for 6 days in the presence of vehicle, 10 μM RU-SKI 43 alone or in combination with indicated concentrations of 4-hydroxytamoxifen (4-OH Tam). Cell numbers were quantified and normalized to vehicle treated cells (100 x (drug/vehicle). Bars represent mean ± SD (n = 3) for all panels. Three independent experiments were performed in duplicate using cells at three different passages. *P ≤ 0.05; **P ≤ 0.01; ***P ≤ 0.001; ****P ≤ 0.0001; Student’s *t* test.

Tamoxifen is the most widely used hormone therapy for breast cancer [5]. We therefore investigated whether RU-SKI 43 could enhance the ability of tamoxifen to reduce ER positive cell proliferation. Combined treatment with RU-SKI 43 and 4-hydroxytamoxifen (4-OH Tam) significantly reduced proliferation in T47D, HCC1428, and MCF7 cells compared to either drug alone (Figure 9A-C). We then tested whether tamoxifen resistant cells retained sensitivity to Hhat inhibition. BT474 cells are tamoxifen resistant (Figure 9D) due to HER2 amplification, but exhibited reduced proliferation after Hhat knockdown (Figures 1F and 2D) or inhibition (Figures 3B, 7A and 9D). We next examined the effect of Hhat inhibition in cells that acquire tamoxifen resistance in the absence of HER2 amplification. We used a tamoxifen resistant clone, TamR, generated by culturing MCF7 cells in the presence of 10^−7^ M 4-OH Tam [56], and verified that this clone does not have HER2 amplification (Additional file 1: Figure S1). Depletion of Hhat in TamR cells (Additional file 2: Figure S2F) significantly decreased cell proliferation (Figure 1G). In addition, treatment of TamR cells with RU-SKI 43 reduced cell proliferation by 60%, similar to the effect observed in MCF7 cells (Figure 9C,E). ER activation in TamR cells was not altered in the presence of RU-SKI 43 (Figure 4C). Furthermore, the combination of RU-SKI 43 and tamoxifen led to a more potent inhibition of TamR proliferation (Figure 9E) compared to RU-SKI 43 treatment alone. Taken together, these data suggest that Hhat can serve as a target in cells that acquire tamoxifen resistance through either HER2 amplification or other mechanisms.

## Discussion

In this study, we used genetic and pharmacologic methods to establish Hhat as a critical regulator of breast cancer cell growth. Hhat depletion or treatment with the selective Hhat inhibitor RU-SKI 43 reduced both anchorage-dependent and anchorage-independent proliferation of ER positive cells (Figures 1, 2 and 3). Hhat knockdown or inhibition also reduced the growth of HER2 positive and tamoxifen resistant cells (Figures 1, 7 and 9). Inhibition of breast cancer cell growth by RU-SKI 43 was dose dependent and was rescued by Hhat overexpression (Figure 3). Treatment with C2, a compound that is structurally similar to RU-SKI 43 but does not inhibit Hhat activity [45], had no effect on proliferation (Figure 3). We have previously demonstrated that the inhibitory effect of RU-SKI 43 is selective for Hhat, as this compound does not inhibit palmitoylation of H-Ras and Fyn, myristoylation of c-Src, or fatty acylation of Wnt3a by Porcupine, another member of the MBOAT family [45]. Overexpressing increasing amounts of Hhat, but not Porcupine, decreases the inhibitory effect of RU-SKI 43 on Shh palmitoylation [45]. Moreover, overexpression of Hhat reduced the inhibitory effect of RU-SKI 43 on breast cancer cell proliferation (Figure 3H). It is possible that breast tumors that overexpress Hhat due to gene amplification might require higher doses of Hhat inhibitor. However, our finding that RU-SKI 43 inhibits the growth of T47D cells, which express relatively high levels of Hhat compared to other cell lines (Figure 1A), suggests that Hhat inhibition is a viable approach to reducing breast cancer cell growth. Taken together, these data suggest that the primary target of RU-SKI 43 is Hhat, and provide the first identification of Hhat as a novel target in breast cancer.

Hhat was identified as the palmitoyl acyltransferase for Shh and the hedgehog family of proteins [36,40], and Hhat inhibition has been shown to block Shh signaling [45]. Thus, it was important to monitor expression of Shh and hedgehog signaling pathway components in breast cancer cells. There is general agreement between the findings reported here and in four other studies [17,19-21] that examined expression levels of Shh pathway components in four of the same cell lines (T47D, MCF7, MDA-MB-231, and BT474) that we analyzed: 1) Shh is expressed in MCF7, T47D, and MDA-MB-2312) Ptch-1 and 2 are expressed in all four cell lines, and 3) Smo is expressed in T47D and BT474 but not in MCF7 and MDA-MB-231 cells. However, in contrast to other studies, we did not detect Ihh, Dhh, Gli-1 or Gli-2 expression in MCF7 or T47D cells (Figure 5, Additional file 3: Figure S3). Differences in Gli expression among the four studies may be due to differences in culture methods or confluence state of cells.

Our study addresses two key questions regarding the role of Shh in breast cancer: 1) Do Shh expressing cells exhibit an autocrine response to Shh? 2) If so, does this occur through canonical or non-canonical signaling? Here, we identify two cell lines, T47D and HCC1428, where knockdown of Shh reduced anchorage dependent and independent proliferation (Figure 5). T47D cells can also undergo increased proliferation in response to exogenous Shh, but this increase is only evident after endogenous levels of Shh are depleted (Figure 6). However, T47D and HCC1428 cells neither express Gli-1 (Figure 5) nor respond to treatment with the Smo inhibitor LDE-225 (Figure 6), indicating the presence of non-canonical Shh signaling. Others have also noted that treatment with cyclopamine, a Smo inhibitor, reduces proliferation of certain breast cancer cells, but that this does not correlate with Smo expression [19] or inhibition [20]. In this study, we used LDE-225 at 0.1 μM, a concentration 100x higher than IC_50_ for binding of LDE-225 to Smo [54], and found no effect on proliferation of any of the breast cancer cells (Figure 6). Taken together, these findings suggest that in breast cancer cells, canonical Smo mediated signaling is not operative, and cells that respond to Shh do so via non-canonical, Smo-independent signaling. This conclusion is supported by multiple recent studies documenting the existence of non-canonical, Smo-independent Shh signaling pathways in normal and cancer cells [21,41,57-59].

The findings presented here indicate that Hhat has regulatory roles in addition to Shh signaling. Shh depleted cells were still sensitive to Hhat inhibition and this growth defect was not rescued by addition of exogenous Shh (Figure 6). Moreover, we demonstrate a requirement for Hhat, but not Shh, for proliferation of multiple ER positive cells (Figures 1, 2 and 5), consistent with our recent report showing that Hhat can have Shh-independent functions in pancreatic cancer cells [41]. We speculate that Hhat has substrates in addition to the hedgehog family. Studies in flies have shown that the EGF-like ligand Spitz is a substrate for Rasp, the *Drosophila melanogaster* ortholog of Hhat [60]. Although no Spitz ortholog has been identified in mammals, and none of the mammalian EGF family ligands appear to be palmitoylated by Hhat, our findings of hedgehog-independent roles of Hhat suggest that other substrates exist. We conclude that Hhat can promote breast cancer cell growth in a Shh independent manner.

All ER positive cell lines that we tested responded to Hhat depletion or inhibition by exhibiting decreased proliferation, while triple negative cell lines did not. Multiple lines of evidence argue against the possibility that Hhat operates via a direct, ER-dependent mechanism. First, despite reports that ERα is palmitoylated, ERα is unlikely to be a direct substrate for Hhat. The active site of Hhat is oriented towards the lumen of the endoplasmic reticulum. Hhat mediated palmitoylation occurs in the ER lumen and Hhat only palmitoylates secreted proteins [36,60]. In contrast, ERα is localized to the nucleus, cytosol and plasma membrane, and palmitoylation of ERα is thought to occur in the cytoplasm [47]. Thus, Hhat could not topologically access ERα as a substrate as ERα does not enter the secretory pathway. Second, using ^125^I-iodopalmitate, a sensitive and robust probe for palmitoylated proteins, we were unable to detect incorporation of ^125^I-iodopalmitate into either endogenous or overexpressed ERα. Third, RU-SKI 43 treatment did not alter the localization or activation of ERα, suggesting RU-SKI 43 does not directly affect ERα function (Figure 4). Fourth, depletion or inhibition of Hhat can also inhibit the growth of HER2 positive cells that are ER negative (Figure 7, Additional file 5: Figure S5B), indicating that, in the context of HER2 amplification, Hhat can modulate cell proliferation independently of ER status.

Increased PI3K/mTOR signaling occurs in up to a quarter of breast cancers [13] and upregulation of Akt signaling is associated with resistance to both endocrine and HER2 targeted therapies [12,13]. We observed that simultaneous inhibition of PI3K/mTOR and Hhat led to a greater decrease in cell proliferation than with either agent alone (Figure 8). Similarly, combined treatment with the Hhat inhibitor and tamoxifen was more effective than either drug alone (Figure 9). In addition, we noted that tamoxifen resistant cells, either through HER2 amplification (BT474) or other mechanisms (TamR), maintained sensitivity to Hhat knockdown or inhibition (Figures 1 and 9). Of note, combined treatment of the TamR cells with the Hhat inhibitor and tamoxifen was more effective than RU-SKI 43 alone (Figure 9). Since RU-SKI 43 did not alter ERα activation in TamR cells (Figure 4C), it is possible that other pathways induced during selection for tamoxifen resistance may contribute to the increased sensitivity in this clone. As with all pharmacologic approaches, we cannot exclude the possibility that off-target effects of RU-SKI 43, yet to be identified, contribute to the response in TamR cells. Taken together, these data underscore the therapeutic potential of using Hhat inhibitors alone or in combination with PI3K/mTOR inhibitors or ER modulators to treat breast cancer and circumvent or delay resistance to current treatments.

## Conclusions

In this study, we used cell lines that represent the heterogeneity of breast cancers to establish that Hhat regulates the proliferation of ER positive, HER2 positive, and tamoxifen resistant breast cancer cells. Smo-dependent canonical Shh signaling is not operative in any of the cell lines we tested, and evidence is presented to indicate that Hhat can regulate breast cancer cell growth independently of Shh. Our findings identify Hhat as a novel target for therapeutic intervention in endocrine sensitive and insensitive disease. Together with recent reports of the importance of Hhat in pancreatic and lung cancers [41-44], this study highlights the potential of Hhat inhibitors for therapeutic intervention in human malignancies.

## Methods

### Reagents and antibodies

Lipofectamine 2000® and TRIzol® were obtained from Invitrogen (Carlsbad, CA). Polybrene was purchased from Santa Cruz Biotechnology Inc. (Santa Cruz, CA). Anti-HA antibodies, 17β-estradiol, 4-hydroxytamoxifen, and puromycin were purchased from Sigma (St. Louis, MO). Anti-actin was purchased from BD Bioscience (San Jose, CA). The ErbB2/HER2, ERα, and pSer118 ERα antibodies were purchased from Cell Signaling (Danvers, MA). LDE-225, LY2940002, and lapatinib ditosylate were purchased from Selleckchem (Houston, TX). Rapamycin was obtained from Fisher Scientific (Waltham, MA). Blasticidin S Hydrochloride was obtained from MP Biomedicals (Santa Ana, CA). 0.4% Trypan Blue Solution was purchased from Cellgro (Manassas, VA). Recombinant human Shh(C24II) was purchased from R&D Systems (Minneapolis, MN).

### Plasmids

Plasmids encoding short hairpin RNA (shRNA) sequences for Shh (Clone IDTRCN0000033304), Hhat shRNA 1 (Clone ID TRCN0000035600) and Hhat shRNA 2 (Clone ID TRCN0000035601), cloned into the pLKO.1 vector, were purchased from Open Biosystems (Lafayette, CO). Control pLKO.1 vector, carrying a scrambled shRNA sequence, as well as pHRD8.2 and pCMV VSV-G plasmids, were gifts from Dr. Filippo Giancotti (Memorial Sloan Kettering Cancer Center, New York, NY). The pLenti6/V5-GW/lacZ vector was purchased from Invitrogen (Carlsbad, CA).

### Cell culture

Human breast cancer cell lines were gifts from the following colleagues at Memorial Sloan Kettering Cancer Center, New York, NY: T47D, HCC1428, BT474 (Dr. Jacqueline Bromberg), MCF7 (Dr. Michael Overholtzer), BT549 and MDA-MB-231 (Dr. Alan Hall), Hs578t, CAMA-1, MDA-MB-453, and SK-BR-3 (Dr. Filippo Giancotti). Cells were grown following ATCC guidelines. TamR cells were a gift from Dr. Guangdi Wang (Xavier University of Louisiana, New Orleans, LA) and grown in ATCC-formulated Dulbecco’s Modified Eagle’s Medium, supplemented with 10% FBS and 1.0 × 10^−4^ M 4-hydroxytamoxifen. All cell lines were authenticated by the ATCC/Promega Cell Line Authentication Service using Short Tandem Repeat profiling analysis performed on July 1, 2014. All cell lines were scored as an exact match for the corresponding ATCC human cell line except for the TamR cell line, which was a 93% match to parental MCF7 cells.

### Lentivirus production and knockdown

Endogenous Shh or Hhat were depleted using shRNA delivered to cells via a lentiviral system. Target sequences are: Shh shRNA(CTACGAGTCCAAGGCACATAT), control scrambled shRNA(CCTAAGGTTAAGTCGCCCTCG), Hhat shRNA 1 (GCCACATGGTAGTGTCTCAAA) and Hhat shRNA 2 (CGTGAGCACCATGTTCAGTTT). The shRNA-expressing lentiviruses were produced by co-transfecting confluent 293 T cells in 15 cm plates with the pLKO.1 shRNA plasmid, the HIV packaging vector pHRD8.2, and pcDNA3.1 VSV-G, using Lipofectamine2000®. Virus was collected 48 and 72 h later as follows. First, media was cleared of debris by centrifugation at 500xg for 5 min. Next, the supernatant was filtered through a 0.45 μm filter, and centrifuged at 38720 × g for 2 h at 4°C in SS-34 Rotor on RC6C centrifuge (Sorvall, Asheville, NC). Finally, the pelleted virus was resuspended in ATCC-formulated Dulbecco’s Modified Eagle’s Medium, supplemented with 10% FBS, and stored at −80°C. Transduction of cells with lentiviruses was carried out in the presence of 6 μg/ml Polybrene. Stable cell lines were produced by transducing target cells with either control scrambled, Shh, or Hhat shRNA expressing lentiviruses, followed by selection in puromycin.

### Hhat overexpression

The pLenti6/V5-GW/lacZ vector was purchased from Invitrogen. The lacZ gene was removed by digestion with SpeI and XhoI, and HhatHA flanked by SpeI and XhoI sites was ligated into the vector. All constructs were confirmed by DNA sequencing. Lentivirus was produced as above and stable cell lines were generated by transducing target cells with either LacZ or HhatHA expressing lentiviruses. Cells were selected in Blasticidin S.

### Anchorage dependent cell proliferation

Cells were plated in 6-well plates (0.5-1 × 10^5^ cells/well, depending on cell type). For experiments involving drug treatment, drugs were added to the media 24 h after plating and media was refreshed every 48 h. Cells were grown for up to 6 days, trypsinized and counted with a hemocytometer.

### Anchorage independent cell proliferation

Cells were plated in Corning Costar Ultra-Low attachment 24-well plates (0.1-0.2 × 10^5^ cells/well). For experiments involving drug treatment, drugs were added to the media 24 h after plating and replenished every 48 h. After 14 days, cells were pelleted, washed with PBS, and treated with 0.05% Trypsin-EDTA. The trypsin was quenched with cell culture media, 0.4% Trypan Blue Solution was added and cells were counted with a hemocytometer.

### qRT-PCR

Total RNA was isolated using TRIzol extraction. cDNA was synthesized using the iScript™ cDNA Synthesis Kit (Bio-Rad Laboratories, Hercules, CA) following the manufacturer’s instructions. qRT-PCR was used to determine expression levels of Hhat, Shh, Ihh, Dhh, Patched-1, Patched-2, hHIP, Smoothened, Gli-1, Gli-2, Gli-3 and HPRT using SsoAdvanced™ SYBR® Green Supermix and the CFX Connect Real Time System (Bio-Rad Laboratories, Hercules, CA). Gene specific primers are listed in Additional file 6: Table S1. Hypoxanthine Phosphoribosyltransferase 1 (HPRT) was used as an endogenous reference, and the relative expression levels of each gene were normalized using the comparative Ct method. Gene expression was normalized to the endogenous reference given by 2^−ΔΔCT^.

### Immunoblot analysis

Cells were lysed in radioimmune precipitation assay (RIPA) buffer (150 mM NaCl, 50 mM Tris (pH 7.4), 1% Triton X-100, 0.5% sodium deoxycholate, 0.1% SDS, and 1 mM EDTA). Lysates in sample buffer were electrophoresed on SDS-PAGE gels, transferred to PVDF membranes, and probed with the indicated antibodies. To monitor phosphorylation of ERα Ser118, MCF7 or TamR cells were treated with either DMSO or 10 μM RU-SKI 43 for 4 h. Media was also supplemented with either ethanol or 200 nM 17β-estradiol for the last 30 min of incubation. Cells were lysed in RIPA buffer containing Halt Protease Inhibitor Cocktail and Halt Phosphatase Inhibitor Cocktail (Thermo Scientific). Lysates in sample buffer were electrophoresed on SDS-PAGE gels, transferred to PVDF membranes, and probed with the indicated antibodies.

### Indirect Immunofluorescence

MCF7 cells were seeded onto coverslips in 6-well plates and cultured for an additional 24 h. Cells were treated with either DMSO or 10 μM RU-SKI 43 for 4 h. Cells were fixed with 4% paraformaldehyde for 20 min and permeabilized with 0.2% Triton X-100 for 5 min at room temperature. Cells were incubated with anti-ERα (Cell Signaling) for 1 h followed with incubation with a secondary antibody (Alexa Flour® 488-conjugated anti-mouse IgG) for 45 min. Slides were mounted with ProLong® Gold Antifade (Invitrogen). Images were collected using a Leica SP5 confocal microscope and analyzed with the Leica Application Suite software. Images were collected using the same conditions on the same day ensuring fair side-by-side comparison.

## Additional files

Additional file 1: Figure S1. ER and HER2 expression in breast cancer cell lines. Cell lysates from indicated breast cancer cells were analyzed directly by Western blotting for ER and HER2 expression. The experiment was performed three times using cells at three different passages. Additional file 2: Figure S2. Hhat knockdown in breast cancer cells. **A-I**, T47D **(A)**, MCF **(B)**, HCC1428 **(C)**, CAMA-1 **(D)**, BT474 **(E)**, TamR **(F)**, MDA-MB-231 **(G)**, BT549 **(H)**, and Hs578t **(I)** cells were transduced with either control scrambled or two different Hhat shRNA expressing lentiviruses and selected in puromycin. qRT-PCR was performed to determine the relative expression of Hhat mRNA. Bars represent mean ± SD (n = 3) for all panels. Three independent experiments were performed in duplicate using cells at three different passages. **P* ≤ 0.05; ***P* ≤ 0.01; ****P* ≤ 0.001; *****P* ≤ 0.0001; Student’s *t* test. Additional file 3: Figure S3. Hedehog pathway expression in breast cancer cells. **A-F**, expression of **(A)** Ihh, **(B)** Dhh, **(C)** Ptch-2, **(D)** Gli-2, **(E)** Gli-3, and **(F)** hHIP mRNAs in indicated breast cancer cell lines and a control cervical cancer (HeLa) cell line, was measured by qRT-PCR. Expression of individual genes is shown relative to the expression in HeLa cells, which is set to 1. Bars represent mean ± SD (n = 3). Experiments were performed twice in triplicate. Additional file 4: Figure S4. Shh knockdown in breast cancer cells. **A-E**, T47D **(A)**, MCF **(B)**, HCC1428 **(C)**, BT474 **(D)**, and MDA-MB-231 **(E)** cells were transduced with either control scrambled or Shh shRNA expressing lentiviruses and selected in puromycin. qRT-PCR was performed to determine the fold change in Shh expression. **F-J**, MDA-MB-231 cells were transduced with either control scrambled or Shh shRNA expressing lentiviruses and selected in puromycin. qRT-PCR was performed to determine the fold change in Ihh **(F)** and Dhh **(G)** expression. For B, D, E Bars represent mean ± SD (n = 3). Experiments were performed three times in triplicate. For panels B, D, and E Bars represent mean ± SD (n = 3). Three independent experiments were performed in triplicate using cells at three different passages. For panels A, C, F, and G, bars represent mean ± SD (n = 2). Two independent experiments were performed in triplicate using cells at two different passages. **P* ≤ 0.05; ***P* ≤ 0.01; ****P* ≤ 0.001; *****P* ≤ 0.0001; Student’s *t* test. Additional file 5: Figure S5. Hhat depletion reduces proliferation of MDA-MB-453 cells. A, MDA-MB-453 and SK-BR-3 cells were cultured for 6 days in the presence of DMSO or 5 μM C2. Cell numbers were quantified and normalized to DMSO treated cells (100 x (drug/DMSO). Bars represent mean ± SD (n = 3). Three independent experiments were performed in duplicate using cells at three different passages. B, MDA-MB-453 cells were transduced with either control scrambled or two different Hhat shRNA expressing lentiviruses and selected in puromycin. qRT-PCR was performed to determine the fold change in Hhat expression. Bars represent mean ± SD (n = 3). Three independent experiments were performed in duplicate using cells at three different passages. C, MDA-MB-453 cells stably expressing scrambled or Hhat shRNAs were seeded at 7x10^4^ cells/well in 6-well plates and cell numbers were quantified on day 6. Bars represent mean ± SD (n = 3). Three independent experiments were performed in duplicate using cells at three different passages. **P* ≤ 0.05; ***P* ≤ 0.01; ****P* ≤ 0.001; *****P* ≤ 0.0001; Student’s *t* test. Additional file 6: Table S1. Primers used for qRT-PCR. Primers used for qRT-PCR for indicated genes.

## Abbreviations

Hhat: Hedgehog acyltransferase
Shh: Sonic Hedgehog
ER: Estrogen receptor
HER2: Human epidermal growth factor 2
Smo: Smoothened
Ptch-1: Patched-1
DCIS: Ductal carcinoma *in situ*
IDC: Invasive ductal carcinoma
4-OH Tam: 4-hydroxytamoxifen
MBOAT: Membrane bound O-acyltransferase
