# Supplementary figures and images for: Hedgehog Acyltransferase as a target in estrogen receptor positive, HER2 amplified, and tamoxifen resistant breast cancer cells
Source: Mol Cancer. 2015 Apr 1;14:72. doi: 10.1186/s12943-015-0345-x (PMC4711017; doi:10.1186/s12943-015-0345-x)

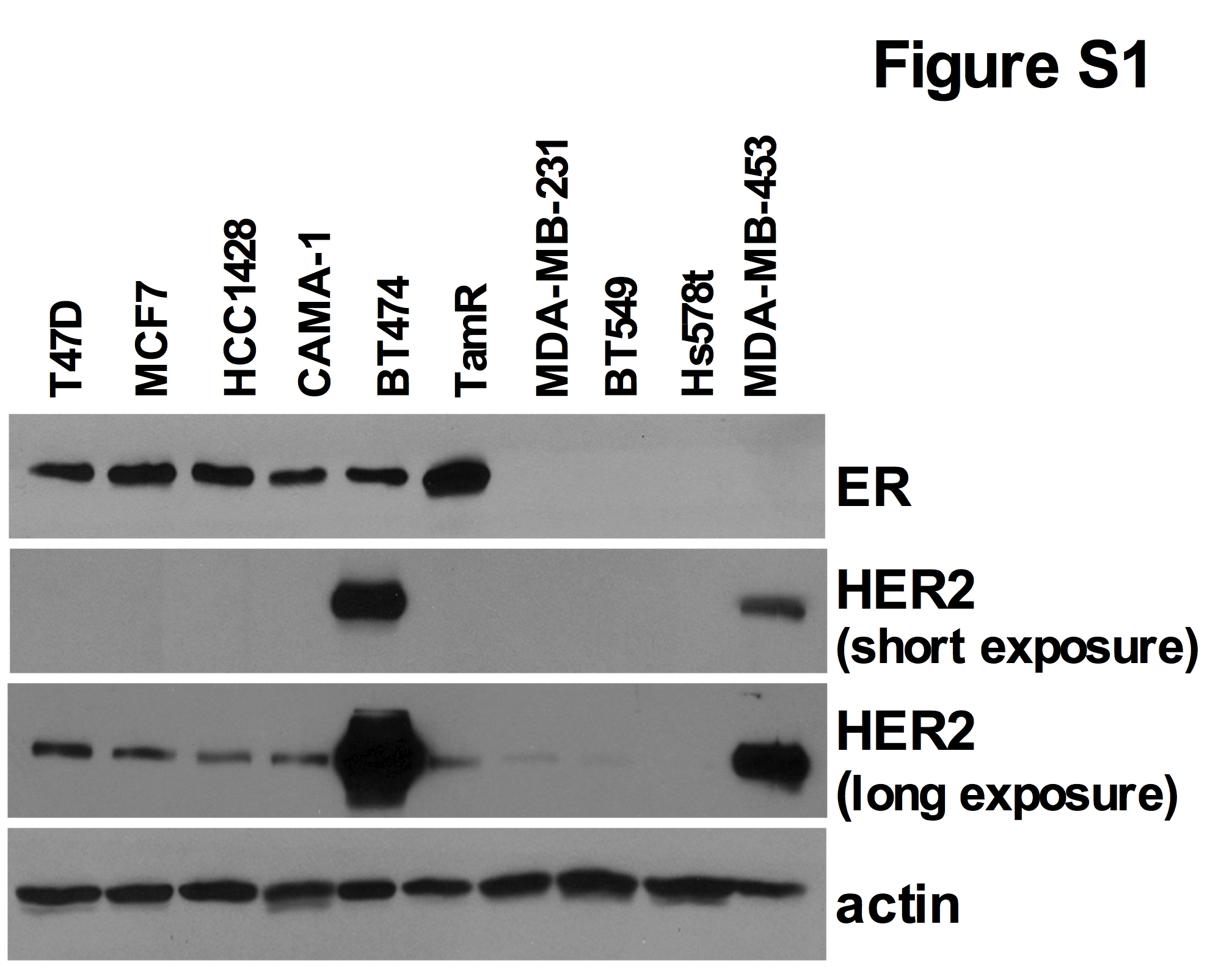

Supplement: Additional file 1: Figure S1. — ER and HER2 expression in breast cancer cell lines. Cell lysates from indicated breast cancer cells were analyzed directly by Western blotting for ER and HER2 expression. The experiment was performed three times using cells at three different passages. [file 12943_2015_345_MOESM1_ESM.tiff]

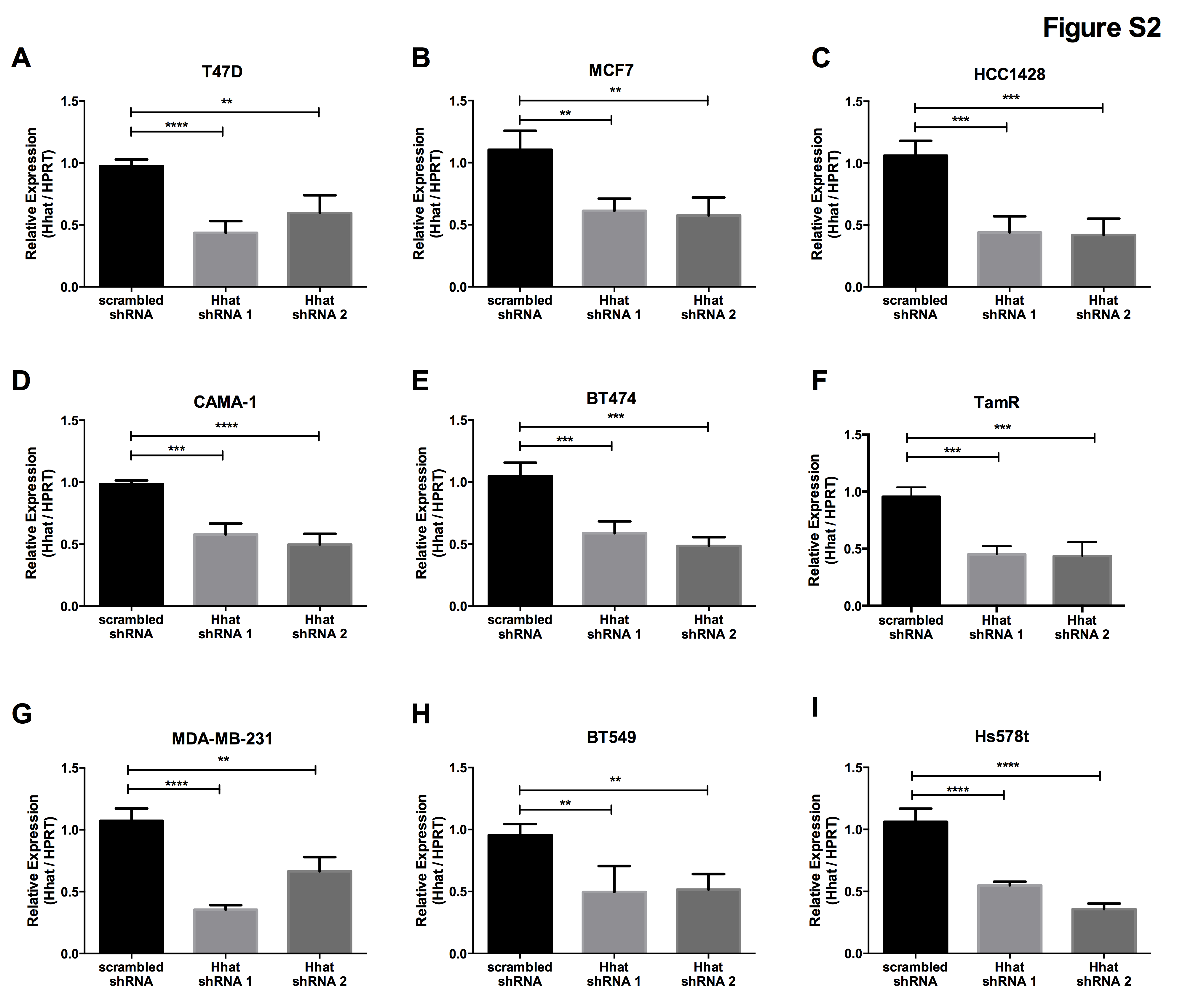

Supplement: Additional file 2: Figure S2. — Hhat knockdown in breast cancer cells. A-I, T47D (A), MCF (B), HCC1428 (C), CAMA-1 (D), BT474 (E), TamR (F), MDA-MB-231 (G), BT549 (H), and Hs578t (I) cells were transduced with either control scrambled or two different Hhat shRNA expressing lentiviruses and selected in puromycin. qRT-PCR was performed to determine the relative expression of Hhat mRNA. Bars represent mean ± SD (n = 3) for all panels. Three independent experiments were performed in duplicate using cells at three different passages. *P ≤ 0.05; **P ≤ 0.01; ***P ≤ 0.001; ****P ≤ 0.0001; Student’s t test. [file 12943_2015_345_MOESM2_ESM.tiff]

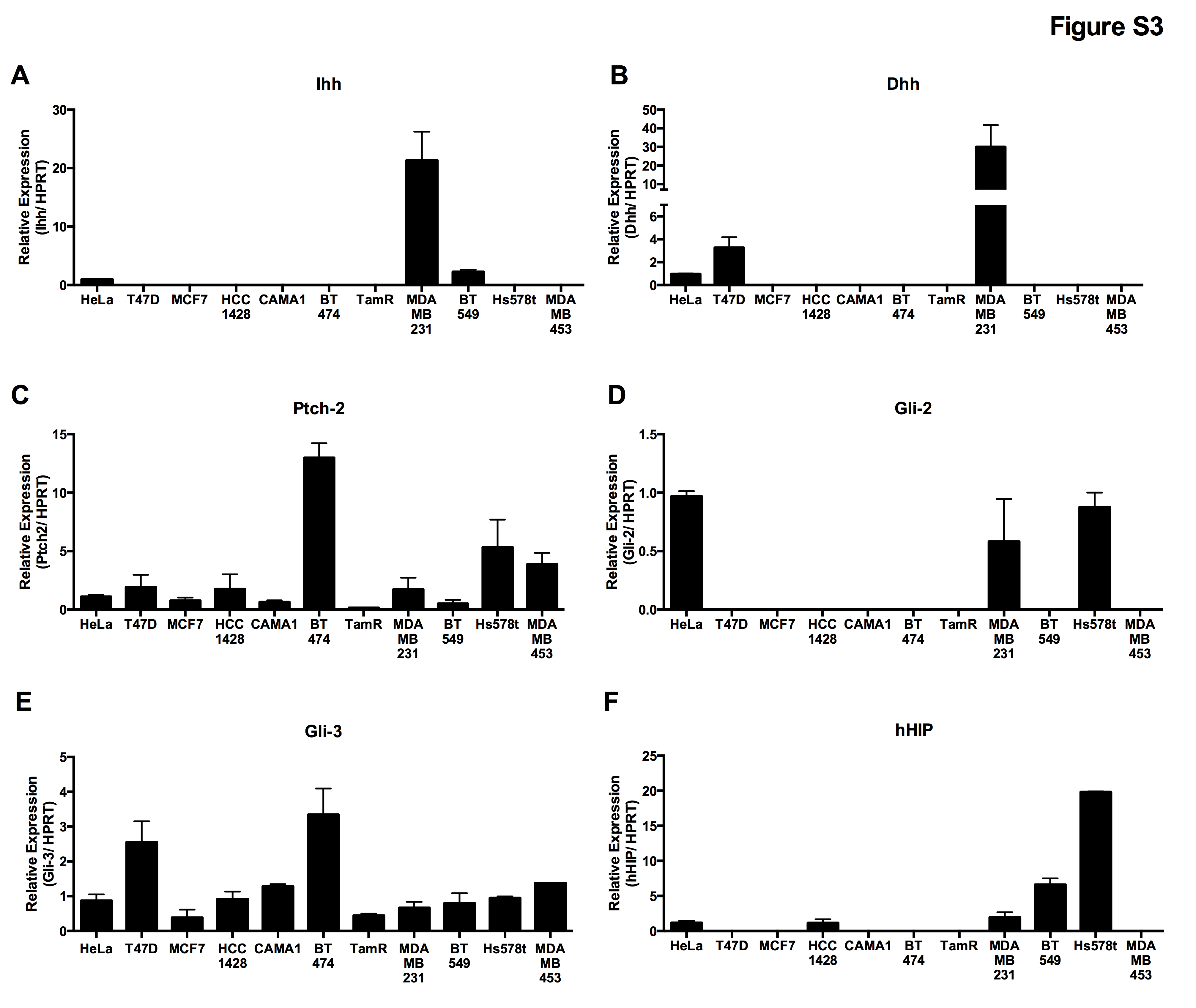

Supplement: Additional file 3: Figure S3. — Hedehog pathway expression in breast cancer cells. A-F, expression of (A) Ihh, (B) Dhh, (C) Ptch-2, (D) Gli-2, (E) Gli-3, and (F) hHIP mRNAs in indicated breast cancer cell lines and a control cervical cancer (HeLa) cell line, was measured by qRT-PCR. Expression of individual genes is shown relative to the expression in HeLa cells, which is set to 1. Bars represent mean ± SD (n = 3). Experiments were performed twice in triplicate. [file 12943_2015_345_MOESM3_ESM.tiff]

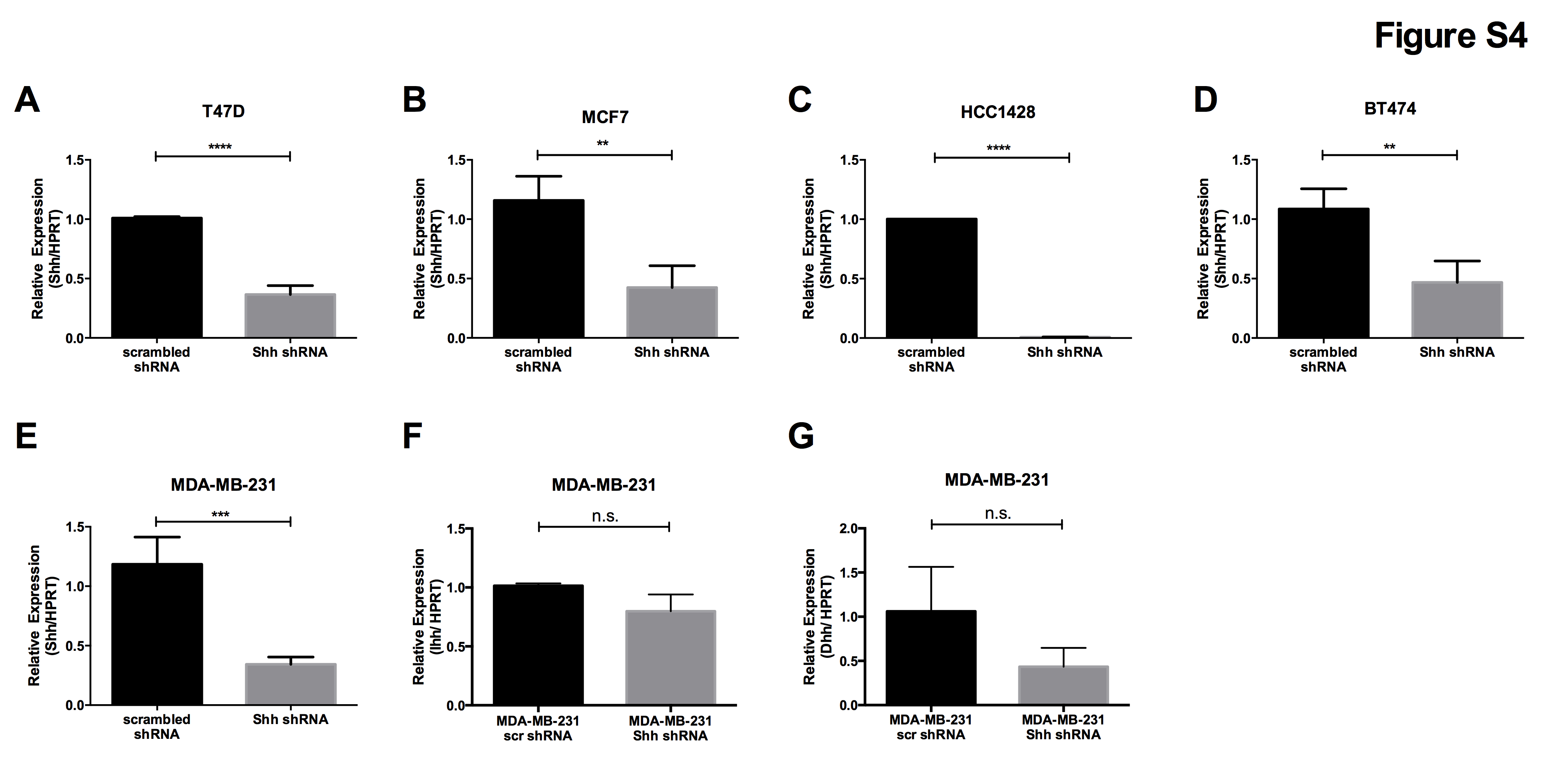

Supplement: Additional file 4: Figure S4. — Shh knockdown in breast cancer cells. A-E, T47D (A), MCF (B), HCC1428 (C), BT474 (D), and MDA-MB-231 (E) cells were transduced with either control scrambled or Shh shRNA expressing lentiviruses and selected in puromycin. qRT-PCR was performed to determine the fold change in Shh expression. F-J, MDA-MB-231 cells were transduced with either control scrambled or Shh shRNA expressing lentiviruses and selected in puromycin. qRT-PCR was performed to determine the fold change in Ihh (F) and Dhh (G) expression. For B, D, E Bars represent mean ± SD (n = 3). Experiments were performed three times in triplicate. For panels B, D, and E Bars represent mean ± SD (n = 3). Three independent experiments were performed in triplicate using cells at three different passages. For panels A, C, F, and G, bars represent mean ± SD (n = 2). Two independent experiments were performed in triplicate using cells at two different passages. *P ≤ 0.05; **P ≤ 0.01; ***P ≤ 0.001; ****P ≤ 0.0001; Student’s t test. [file 12943_2015_345_MOESM4_ESM.tiff]

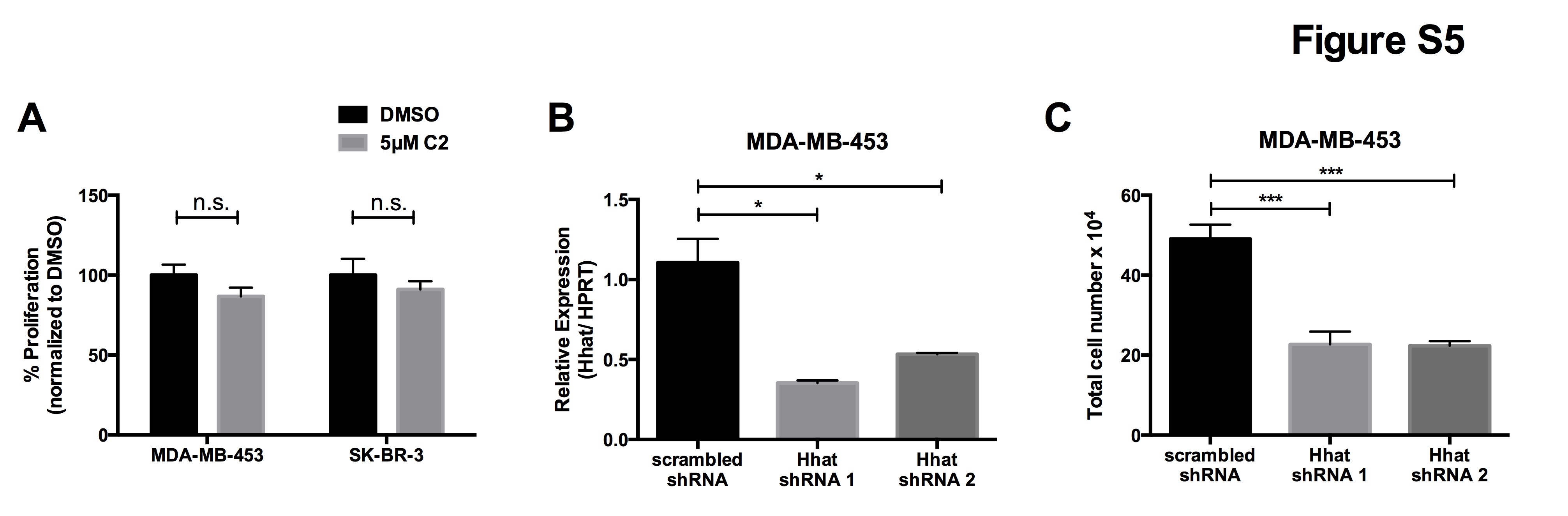

Supplement: Additional file 5: Figure S5. — Hhat depletion reduces proliferation of MDA-MB-453 cells. A, MDA-MB-453 and SK-BR-3 cells were cultured for 6 days in the presence of DMSO or 5 μM C2. Cell numbers were quantified and normalized to DMSO treated cells (100 x (drug/DMSO). Bars represent mean ± SD (n = 3). Three independent experiments were performed in duplicate using cells at three different passages. B, MDA-MB-453 cells were transduced with either control scrambled or two different Hhat shRNA expressing lentiviruses and selected in puromycin. qRT-PCR was performed to determine the fold change in Hhat expression. Bars represent mean ± SD (n = 3). Three independent experiments were performed in duplicate using cells at three different passages. C, MDA-MB-453 cells stably expressing scrambled or Hhat shRNAs were seeded at 7x10^4^ cells/well in 6-well plates and cell numbers were quantified on day 6. Bars represent mean ± SD (n = 3). Three independent experiments were performed in duplicate using cells at three different passages. *P ≤ 0.05; **P ≤ 0.01; ***P ≤ 0.001; ****P ≤ 0.0001; Student’s t test. [file 12943_2015_345_MOESM5_ESM.tiff]
