# Supplementary material for: Hedgehog Acyltransferase as a target in estrogen receptor positive, HER2 amplified, and tamoxifen resistant breast cancer cells
Source: Mol Cancer. 2015 Apr 1;14:72. doi: 10.1186/s12943-015-0345-x (PMC4711017; doi:10.1186/s12943-015-0345-x)
Supplement: Additional file 6: Table S1. — Primers used for qRT-PCR. Primers used for qRT-PCR for indicated genes. [file 12943_2015_345_MOESM6_ESM.docx]

| **Gene symbol** | **Forward primer** | **Reverse primer** |
| --- | --- | --- |
| HHAT | GGGTGCTTGTTTCTGAGATTTG | GGGTACACTATCCTGTGGTTTC |
| SHH | CAGTGGCCAGGATGAAACT | GGATATGTGCCTTGGACTCG |
| IHH | CGGCTTTGACTGGGTGTATT | GAAAATGAGCACATCGCTGA |
| DHH | TGATGACCGAGCGTTGTAAG | GCCAGCAACCCATACTTGTT |
| PTCH-1 | GGCAGCGGTAGTAGTGGTGTTC | TGTAGCGGGTATTGTCGTGTGTG |
| PTCH-2 | GCACTATTACCGCAACTGGCTAC | TCTCCAGTCTGGATGAGCAGCT |
| SMO | TCGCTACCCTGCTGTTATTC | GACGCAGGACAGAGTCTCAT |
| GLI-1 | GCTTTACTGCAGCCTCGT | CCCAGTACATGCTGGTGGTT |
| GLI-2 | GTCAGAGCCATCAAGACCGAGA | GCATCTCCACGCCACTGTCATT |
| GLI-3 | TCAGCAAGTGGCTCCTATGGTC | GCTCTGTTGTCGGCTTAGGATC |
| hHIP | GCCATTCAGTAATGGTCCTTTGG | TGCCACTGCTTTGTCACAGGAC |
| HPRT | CCTGGCGTCGTGATTAGTGATG | CAGAGGGCTACAATGTGATGGC |

**Table S1. Primers used in qRT-PCR**
